# Current perspectives on paediatric HIV management from the Mexico International Aids Society Conference, 2019

**DOI:** 10.4102/sajhivmed.v20i1.1027

**Published:** 2019-10-31

**Authors:** Mohendran Archary, Lee Fairlie, Amy Slogrove

**Affiliations:** 1 Department of Infectious Diseases, University of KwaZulu-Natal, Durban, South Africa; 2 Department of Paediatrics & Child Health, University of KwaZulu-Natal, Durban, South Africa; 3 Wits Reproductive Health and HIV Institute (WRHI), University of the Witwatersrand, Johannesburg, South Africa; 4 Department of Paediatrics and Child Health, Stellenbosch University, Cape Town, South Africa

**Keywords:** 2019 Paediatric ART Conferences, International Aids Society, management of pregnant and breastfeeding women, paediatric/adolescent HIV, treatment update

## Abstract

While acknowledging the great achievements in getting 23 million people living with HIV to access antiretroviral treatment (ART) globally, there is still more to do in order to close the HIV treatment gap between the paediatric and adult ART programmes, with only 54% of children accessing ART compared to 62% of adults. Furthermore, while tremendous global gains have been made in preventing perinatal and postnatal HIV acquisition, HIV-exposed and uninfected children are still not achieving early childhood developmental outcomes comparable to HIV-unexposed children. In this article, we present highlights from two pre-conference meetings (11th International Workshop on HIV Pediatrics and 5th Workshop on Children and Adolescents HIV Exposed and Uninfected) and the International Aids Society (IAS) meeting held in Mexico in July 2019.

## International paediatric HIV workshop

The global scale-up of antiretroviral treatment (ART) and prevention of mother-to-child transmission (PMTCT) strategies has resulted in a dramatic reduction in new paediatric infections. Despite this, globally there were 160 000 new paediatric infections in 2018. This annual meeting provides a good summary of the current and future directions in the prevention and treatment of paediatric and adolescent HIV and co-infections. The key findings presented at this meeting can be categorised into the following: (1) treatment and management of paediatric and adolescent HIV; and (2) management of pregnant and breastfeeding women and PMTCT.

### Treatment and management of paediatric/adolescent HIV

Difficulties in administering medication to children and attendant adherence issues have resulted in poorer virological suppression rates in children than adults.^1^ Several advances in drug formulations and delivery methods were presented at the meeting. Two of the notable new molecules with a potential impact on management of children in the future are GS-6207 and MK-8591.

GS-6207 is the first in the class of capsid inhibitors and is unique in that it acts on various sites of the HIV lifecycle including capsid disassembly on viral entry and capsid assembly and maturation during production of new viral particles. Initial data on subcutaneous administration in adults support 3-monthly administration. No paediatric studies have been conducted.^2^

The second (MK-8591) also has an unique mechanism of action as a nucleoside reverse transcriptase translocation inhibitor that lacks cross-resistance with other nucleoside reverse transcriptase inhibitors (NRTIs). In addition, the formulation has a very long intracellular half-life that potentially allows for administration of very low dosages and the possibility of being given once weekly. Adult data from a fixed-dose combination of MK-8591/doravirine were presented at the main conference. Animal studies suggest that an implantable formulation may support once-a-year administration.^3^ No paediatric data are available on these new formulations.

Other notable new treatment options included cabotegravir/rilpivirine and extended release broadly neutralising antibodies.^4^

Tenofovir alafenamide (TAF) as the optimal background regimen in children was discussed. The advantage of TAF compared to tenofovir diproxil fumarate (TDF) in paediatrics is the potentially lower incidence of renal toxicity and decreases in bone mineral density. A pooled analysis of paediatric TAF studies showed high rates of virological suppression (> 90%) with no emergence of resistance when combined with an integrase inhibitor.^5^ In addition, results from a study using TAF/FTC/elvitegravir/cobicistat fixed drug combination (FDC) in children over 6 years and 25 kg had no serious adverse events (Grade 3/4 AEs) with high rates of viral suppression between 90% and 100%. Of note is the lack of data on using TAF with rifampin-based therapy in paediatrics; data in adults suggest twice daily administration may overcome this interaction.^6^

Novel delivery methods of ART for treatment and prevention of HIV hold exciting promise for children. Long-acting subcutaneous infusions, injections and implants have shown promise in adults and are moving rapidly into the paediatric arena. Transdermal drug delivery using an adhesive patch with micro-needles was described with data in adults achieving acceptable cabotegravir concentrations with once-monthly application.^7^ This drug delivery method holds several advantages for use in children by allowing flexibility to alter doses in a growing child together with easy application and removal, if required.

Achieving the goals of 90:90:90 requires improved paediatric case finding which was addressed in a plenary talk and several oral abstract presentations. Traditional health facility-based testing does not identify HIV-infected children before the onset of symptoms. Community-based screening and index case testing were suggested as an alternative case finding strategy for early identification.^8^ Untested children often have siblings or parents that have been previously tested, with failure to link other family members into care. Effective community engagement and participation is necessary to facilitate case finding.^9^ In settings with high burden of infections, early infant diagnosis together with testing during immunisation visits was cost-effective.^10^ Getting children to start ART and maintaining viral suppression in order to achieve the last two 90s is challenging, especially in HIV-infected newborns, young infants and adolescents. Stratifying intensity of follow-up, counselling and the package of care based on virologic response to therapy was found to be feasible even in rural settings.^11^ Silent transfers of patients (self or poorly documented transfers) between facilities accounted for 65% of patients that were labelled as being lost to follow-up in a cohort from the Western Cape province, highlighting the challenges with linkage of patients.^12^ Improved data management systems and unique identifiers to track patients were recommended by the authors.

### Management of pregnant and lactating women and prevention of mother-to-child transmission

Eagerly awaited results from the Botswana Tsepamo birth surveillance study on the risk of neural tube defects (NTD) in infants born to mothers receiving dolutegravir (DTG) peri-conception were presented indicating a reduction in the risk from that previously reported, but still above the background NTD risk. In addition, data from the International Antiretroviral Pregnancy Registry (APR) showed an overall risk of NTD following peri-conception ART exposure of 0.03%.^13^ In the registry, there were only 248 peri-conception DTG exposures with 1 NTD, limiting the ability to make definitive conclusions. It was noted that the majority of data for the registry was obtained from the United States/Canada (75%), with only 7% from Africa, and an appeal was made to report peri-conception ART exposure to local pharmacovigilance structures (National Department of Health, National Pharmacovigilance Centre) and international pregnancy registries (www.APRegistry.com or https://globalbirthdefects.tghn.org). Apart from birth defects, infants exposed in utero to HIV and ART had poorer weight-for-age (WAZ) and length-for-age (HAZ) *z*-scores compared to HIV-unexposed infants.^14^

Overall, the workshop highlighted major gains that have been achieved in preventing new paediatric infections and treating HIV-infected children and adolescents. However, to achieve the 90:90:90 goals, new clinical- and community-based strategies are needed to find and treat patient populations that have poor health seeking behaviour and disruptive social networks that make consistent adherence to a treatment regimen difficult.

## International AIDS Society conference

The main IAS conference covered numerous tracks including basic science, clinical science, prevention science and social, behavioural and implementation science.

In this article, we provide feedback on aspects of the main conference that related to children, adolescents and pregnant women, with a focus on optimised HIV treatment and PMTCT.

### Optimised maternal antiretroviral treatment and prevention of mother-to-child transmission

In addition to the eagerly awaited Tsepamo data, presented in the workshop, additional data were presented from the Botswana Ministry of Health and Wellness, showing a prevalence of 0.66% (95% confidence interval [CI] 0.02–3.69) in women conceiving on DTG-based ART.^15^ Although only one additional child enrolled in the Tsepamo trial was noted to develop an NTD when following all trial participants up until conclusion of their pregnancies, this prevalence remains higher than reported in women conceiving on any other ART regimen and HIV-negative women.^16^ Data from Brazil, where 384 women conceived on DTG, showed no NTDs (95% CI 0–0.003).^17^ These updated findings prompted slight wording changes within the World Health Organization (WHO) guidelines, now recommending DTG-based ART as the preferred regimen for all HIV-infected patients, although in women of child-bearing age, an informed decision regarding their regimen and contraception must be made before deciding on a DTG- or efavirenz-based regimen.^18^

The ADVANCE study randomised 1053 adolescents and adults to receiving a DTG-based regimen with either TAF or TDF and emtricitabine (FTC), compared to standard of care efavirenz/FTC/TDF. The study reported non-inferiority for DTG-based regimens at 48 weeks, with the primary endpoint of viral load (VL) < 50 copies/mL in an intention to treat analysis. Toxicity was low overall across the study arms; however, significant weight gain particularly in black South African women occurred in those receiving DTG/TAF/FTC (median 10 kg increase), compared to DTG/TDF/FTC (median 5 kg increase) and EFV/TDF/FTC (median 3 kg increase)^19^ were randomised to DTG/TDF/FTC versus EFV(400 mg)/TDF/FTC.^20^ Although this weight gain or the implications thereof are currently not well understood, further evaluation of this finding is warranted. However, as participants enrolled were relatively well, this raises concern regarding long-term risks for cardiovascular disease in women receiving DTG and TAF combination ART. Sixty-five women conceived on the study with no increased risk for adverse pregnancy outcomes in women receiving DTG at conception.

A study in Johannesburg and Tshwane, South Africa, offered point-of-care maternal VL and early infant diagnosis HIV testing around the time of delivery only during ‘office hours’. Of 1762 valid VLs, around 36.4% were unsuppressed at delivery with a VL > 50 copies/mL). Fortunately, infant HIV infection rates were low (65/4333; 1.5%); however, this highlights gaps in optimised maternal ART coverage and uptake of VL testing at delivery, with potential risk of HIV transmission.^21^

### HIV treatment and treatment outcomes in children and adolescents

As guidelines move to a universal regimen for all HIV-positive persons, children lag behind in the era of DTG. Data were presented from the ODYSSEY trial, regarding the pharmacokinetics (PK) of 5 mg DTG dispersible tablets in 28 children weighing 6 kg to < 20 kg (in three weight bands: 6 kg to < 10 kg, 10 kg to < 14 kg and 14 kg to < 20 kg) from Zimbabwe and Uganda. In the weight bands between 10 kg to < 14 kg and 14 kg to < 20kg, PK data were similar to the published data in adults, older children and younger children. However, as in the 6 to < 10 kg group, some children had low trough concentration (*C*_trough_) levels with high inter-participants variability; further PK data are required for this group and children weighed between 3 kg and < 6 kg, highlighting complexities of HIV treatment in younger age groups.^22^

A study from Zimbabwe analysed results of genotypic resistance testing in 160 of 185 children with virological failure on first and second line regimens, and calculated a total genotypic susceptibility score (tGSS) for a switch to protease inhibitor (PI)- or DTG-based regimens, respectively.^23^ The tGSS demonstrated that therapy with the tenofovir–lamivudine–dolutegravir (TLD) combination tablet may result in DTG monotherapy, due to dual NRTI resistance with associated risks of virologic failure and future DTG resistance.^23^

Data from the International epidemiologic Databases to Evaluate AIDS Southern Africa collaboration (IeDEA-SA) showed that between 2004 and 2017 perinatally HIV-infected children and adolescents had suboptimal retention in care, suboptimal VL suppression rates and mortality, with particular risk for those who initiate ART at older ages and more severe immunosuppression. Adolescents, especially those at high risk, require additional support and follow-up to prevent morbidity and mortality.^24^

## Fifth workshop on children who are HIV-exposed and uninfected

The fifth workshop on children who are HIV-exposed and uninfected (CHEU) was themed around the first 1000 days of life (conception to age 2 years), with presentations spanning basic and clinical science, policy, programmatic and research considerations. According to the Joint United Nations Programme on HIV and AIDS (UNAIDS) estimates, globally there were 14.8 million CHEU (age 0–14 years) in 2018, with 3.5 million (24%) living in South Africa. The prevalence of CHEU exceeded 20% in four southern African countries: Eswatini (32%), Botswana (27%), South Africa (22%) and Lesotho (21%).^25^

### The HIV-exposed *in utero* environment

Untangling the mechanisms of adverse birth outcomes in pregnant women living with HIV (pWLHIV) and the role that HIV, specific antiretroviral drugs or other maternal factors play is crucial to securing optimal outcomes for CHEU. In pWLHIV cohorts on non-PI-based ART in Cape Town, timing of ART initiation either preconception or during pregnancy had no influence on placental pathology. However, T-regulatory cells were significantly lower at birth in CHEU than children HIV unexposed and uninfected (CHUU).^26^ In a Canadian cohort of pWLHIV, PI-based ART was associated with lower progesterone and prolactin levels, altered placental morphology and inefficient or over-worked placentas compared to pWLHIV on non-PI-based ART and pregnant women without HIV.^27^ In a mouse model, normal placental spiral artery remodelling and trophoblast invasion, controlled by progesterone and prolactin, were inhibited by lopinavir/ritonavir but not atazanavir/ritonavir or darunavir/ritonavir.^27^ Further work is needed to determine whether these endocrine and placental alterations are associated with preterm birth and intrauterine growth restriction in pWLHIV.

### Solutions to children who are HIV-exposed and uninfected vulnerabilities in early childhood

In a Belgian cohort of CHEU compared to HIV-unexposed children, neonatal immune parameters as well as infectious morbidity risk differed by timing of initiation of maternal ART.^28^ Infants of mothers on preconception ART had immune and infectious morbidity profiles similar to HIV-unexposed infants, whereas infants of mothers on pregnancy-initiated ART showed alterations in cellular and humoral immunity at birth, predictive of and associated with a threefold higher risk for infectious-cause hospitalisations. In the Zimbabawean SHINE trial, conducted in the context of universal maternal ART and high sustained breastfeeding, CHEU had an almost 50% increased risk of stunting at 18 months than HIV-unexposed children (RR 1.48; 95% CI 1.34–1.64) and mortality at 18 months in children born to WLHIV was almost 40% higher than HIV-unexposed children (RR 1.39; 95% CI 1.02–1.89).^29^ There were also significant deficits in gross motor, fine motor and language development at 24 months in CHEU compared to HIV-unexposed children, with normalisation of neurodevelopment in the CHEU group randomised to both a water/sanitation/hygiene and an infant and young child feeding intervention.^30^

Zambia is rolling out a ‘SmartCare’ system that is optimising electronic medical records held on an individual patient ‘Smart Card’ to facilitate confidential communication and availability of HIV-related information for all people living with HIV and all children who are HIV-exposed.^31^ South Africa is supporting early childhood development through the ‘Side-by-Side’ campaign built around the five pillars of nutrition, love, protection, healthcare and extracare. With almost 25% of children in South Africa being CHEU, a dedicated service and monitoring of CHEU is unfeasible and undesirable.^32^ There is a need however to identify the subset of CHEU at highest risk for poor outcomes and requiring linkage to the fifth pillar of extracare and support. Globally, two policy agendas are converging with synergies between the Start Free Stay Free AIDS Free agenda and the Nurturing Care Framework that can be optimised to achieve improved early childhood development outcomes for all children affected by HIV.^33^

## Conclusion

Remarkable success has been achieved to ensure an HIV-free start to life for over 1 million children born each year to women with HIV. However, investment in more detailed research is required to understand why CHEU are not surviving and thriving as well as children born to women without HIV and how to support families affected by HIV to achieve optimal outcomes for their children. For children who are infected with HIV, improvements in ART and ART drug delivery mechanism hold the promise of simplified treatment regimens.

## References

[1] Davies MA, Moultrie H, Eley B, et al Virologic failure and second-line antiretroviral therapy in children in South Africa – The IeDEA Southern Africa collaboration. J Acquir Immune Defic Syndr. 2011;56(3):270–278. 10.1097/QAI.0b013e3182060610 21107266 PMC3104241

[2] Sager JE, Begley R, Rhee M, et al Safety and PK of subcutaneous GS-6207, a novel HIV-1 capsid inhibitor [homepage on the Internet]. Abstract No. 141. CROI Conference; 2019 Mar 4–7; Seattle, WA [cited n.d.]. Available from: http://www.croiconference.org/sessions/safety-and-pk-subcutaneous-gs-6207-novel-hiv-1-capsid-inhibitor.

[3] Barrett S, Teller R, Forster S, et al Extended-duration MK-8591-eluting implant as a candidate for HIV treatment and prevention. Antimicrob Agents Chemother. 2018;62(10):1–13. 10.1128/AAC.01058-18 PMC6153840 30012772

[4] Rajoli RKR, Back DJ, Rannard S, et al In silico dose prediction for long-acting rilpivirine and cabotegravir administration to children and adolescents. Clin Pharmacokinet. 2018;57(2):255–266. 10.1007/s40262-017-0557-x 28540638 PMC5701864

[5] Cox S, Porter D, White K, Graham H, Pikora C, Callebaut C Tenofovir alafenamide-based regimens: A pooled resistance analysis in pediatric participants [homepage on the Internet]. Oral Abstract Presentations No. 4. 10th International AIDS Society Conference on HIV Science; 2019 Jul 21–24; Mexico City [cited n.d.]. Available from: http://regist2.virology-education.com/presentations/2019/HIVPed/04a_Cox.pdf.

[6] Rakhmanina N, Natukunda E, Kosalaraksa P Safety and efficacy of E/C/F/TAF in virologically suppressed, HIV infected children through 96 weeks [homepage on the Internet]. Short Oral Poster Presentations No. 22. 10th International AIDS Society Conference on HIV Science; 2019 Jul 21–24; Mexico City [cited n.d.]. Available from: http://regist2.virology-education.com/presentations/2019/HIVPed/04a_Cox.pdf.

[7] Rajoli RKR, Curley P, Back D In silico drug interaction of long-acting rilpivirine and cabotegravir with rifampin [homepage on the Internet]. Abstract No. 485. CROI Conference; 2018 Mar 4–7; Boston, MA [cited n.d.]. Available from: https://www.croiconference.org/sessions/silico-drug-interaction-long-acting-rilpivirine-and-cabotegravir-rifampin.

[8] Neary J Untested children reached through index case testing often have previously tested siblings and poor historic PMTCT engagement [homepage on the Internet]. Poster Walk No. 36. 10th International AIDS Society Conference on HIV Science; 2019 Jul 21–24; Mexico City [cited n.d.]. Available from: https://www.virology-education.com/wp-content/uploads/2019/07/11PED_-Program.pdf.

[9] Okorie GS Effective community engagement and participation, key to Nigeria achieving the first 90’ target of eMTCT [homepage on the Internet]. Poster Walk No. 33. 10th International AIDS Society Conference on HIV Science; 2019 Jul 21–24; Mexico City [cited n.d.]. Available from: https://www.virology-education.com/wp-content/uploads/2019/07/11PED_-Program.pdf.

[10] Ciaranello A The clinical impact and cost-effectiveness of routine HIV screening and testing at infant immunization visits in Côte d’Ivoire (CI), South Africa, and Zimbabwe [homepage on the Internet]. Short Oral Poster Presentation No. 21. 10th International AIDS Society Conference on HIV Science; 2019 Jul 21–24; Mexico City [cited n.d.]. Available from: https://www.virology-education.com/wp-content/uploads/2019/07/11PED_-Program.pdf.

[11] Shava A Retention and viral suppression of children and adolescents in HIV care, Zimbabwe [homepage on the Internet]. Poster Walk No. 52. 10th International AIDS Society Conference on HIV Science; 2019 Jul 21–24; Mexico City [cited n.d.]. Available from: https://www.virology-education.com/wp-content/uploads/2019/07/11PED_-Program.pdf.

[12] Nyakato P, Boulle A, Wood R, et al Outcomes of children and adolescents living with HIV considered lost to follow up at IeDEA-SA cohorts in the Western Cape: Linkage to Western Cape Provincial Health Data Centre records [homepage on the Internet]. Oral Abstract Presentation No. 28. 10th International AIDS Society Conference on HIV Science; 2019 Jul 21–24; Mexico City [cited n.d.]. Available from: http://regist2.virology-education.com/presentations/2019/HIVPed/28a_Nyakato.pdf.

[13] Mofensen L, Vannappagari V, Scheuerle AE, et al Periconceptional antiretroviral exposure and central nervous system (CNS) and neural tube birth defects – Data from Antiretroviral Pregnancy Registry (APR) [homepage on the Internet]. Oral Abstract Presentation No. 12. 10th International AIDS Society Conference on HIV Science; 2019 Jul 21–24; Mexico City [cited n.d.]. Available from: http://regist2.virology-education.com/presentations/2019/HIVPed/12a_Mofenson.pdf.

[14] Nyemba DC, Kalk E, Hlengiwe M, et al Lower birth weight-for-age and length-for-age Z-scores in infants with *in utero* HIV and ARV exposure: A prospective study in Cape Town, South Africa [homepage on the Internet]. Oral Abstract Presentation No. 15. 10th International AIDS Society Conference on HIV Science; 2019 Jul 21–24; Mexico City [cited n.d.]. Available from: http://regist2.virology-education.com/presentations/2019/HIVPed/15a_Chiwoniso.pdf.

[15] Zash R, Holmes L, Diseko M, et al Neural tube defects by antiretroviral and HIV exposure in the Tsepamo Study, Botswana [homepage on the Internet]. Oral Abtract Presentation No. MOAX0105LB. 10th International AIDS Society Conference on HIV Science; 2019 Jul 21–24; Mexico City [cited n.d.]. Available from: http://programme.ias2019.org/Abstract/Abstract/4822.

[16] Raesima MM, Forhan S, Thomas V, et al Addressing the safety signal with dolutegravir use at conception: Additional surveillance data from Botswana [homepage on the Internet]. Oral Abtract Presentation No. MOAX0106LB. 10th International AIDS Society Conference on HIV Science; 2019 Jul 21–24; Mexico City [cited n.d.]. Available from: http://programme.ias2019.org/Abstract/Abstract/5089.

[17] Pereira G, Kim A, Jalil E, et al No occurrences of neural tube defects among 382 women on dolutegravir at pregnancy conception in Brazil [homepage on the Internet]. Oral Abtract Presentation No. MOAX0104LB. 10th International AIDS Society Conference on HIV Science; 2019 Jul 21–24; Mexico City [cited n.d.]. Available from: http://programme.ias2019.org/Abstract/Abstract/4991.

[18] World Health Organisation Update of recommendations on first- and second-line antiretroviral regimens – Policy brief [homepage on the Internet]. [cited n.d.]. Available from: https://www.who.int/hiv/pub/arv/arv-update-2019-policy/en/.

[19] Venter WF, Moorhouse M, Sokhela S, et al The ADVANCE trial: Phase 3, randomized comparison of TAF/FTC/DTG, TDF/FTC/DTG or TDF/FTC/EFV for first-line treatment of HIV-1 infection [homepage on the Internet]. Oral Abtract Presentation No. WEAB0405LB. 10th International AIDS Society Conference on HIV Science; 2019 Jul 21–24; Mexico City [cited n.d.]. Available from: https://onlinelibrary.wiley.com/doi/10.1002/jia2.25327.

[20] Hill A, Venter WF, Delaporte E, et al Progressive rises in weight and clinical obesity for TAF/FTC/DTG and TDF/FTC/DTG versus TDF/FTC/EFV: ADVANCE and NAMSAL trials. Oral Abtract Presentation No. MOAX0102LB [homepage on the Internet]. 10th International AIDS Society Conference on HIV Science; 2019 Jul 21–24; Mexico City [cited n.d.]. Available from: https://onlinelibrary.wiley.com/doi/10.1002/jia2.25327.

[21] Moyo F, Haeri Mazanderani A, Murray T, et al Characterizing viral load burden among HIV positive women around time of delivery: Findings from four tertiary obstetric units in Gauteng, South Africa [homepage on the Internet]. Oral Abtract Presentation No. TUAB0104. 10th International AIDS Society Conference on HIV Science; 2019 Jul 21–24; Mexico City [cited n.d.]. Available from: https://onlinelibrary.wiley.com/doi/10.1002/jia2.25327.

[22] Waalewijn H, Bollen PDJ, Moore C Pharmacokinetics of dolutegravir 5 mg dispersible tablets in children weighing 6 to < 20 kg dosed using WHO weight bands [homepage on the Internet]. Oral Abtract Presentation No. WEAB0401LB. 10th International AIDS Society Conference on HIV Science; 2019 Jul 21–24; Mexico City [cited n.d.]. Available from: https://onlinelibrary.wiley.com/doi/10.1002/jia2.25327.

[23] Kouamou VM, Manasa J, McGregor A, et al Dolutegravir containing regimens may need optimization for African youth failing ART [homepage on the Internet]. Oral Abtract Presentation No. WEAB0203. 10th International AIDS Society Conference on HIV Science; 2019 Jul 21–24; Mexico City [cited n.d.]. Available from: https://onlinelibrary.wiley.com/doi/10.1002/jia2.25327.

[24] Tsondai PR, Braithwaite K, Fattii G Describing the characteristics and long-term outcomes of adolescents living with perinatally acquired HIV in the IeDEA-Southern Africa collaboration: 2004 to 2017 [homepage on the Internet]. Oral Abtract Presentation No. WEAB0201. 10th International AIDS Society Conference on HIV Science; 2019 Jul 21–24; Mexico City [cited n.d.]. Available from: https://onlinelibrary.wiley.com/doi/10.1002/jia2.25327.

[25] Joint United Nations Programme on HIV/AIDS 2019 UNAIDS estimates [homepage on the Internet]. [cited n.d.]. Available from: http://aidsinfo.unaids.org.

[26] Gray C The impact of maternal HIV infection on placental and neonatal immunology among children HIV exposed uninfected [homepage on the Internet]. Oral Presentation. 5th Workshop on Children and Adolescents HIV Exposed and Uninfected; 2019 Jul 21–24; Mexico City [cited n.d.]. Available from: https://www.iasociety.org/HIV-Programmes/Programmes/Paediatrics-CIPHER/5th-HIV-Exposed-Uninfected-Child-and-Adolescent-Workshop.

[27] Serghides L ARV exposure and placental morphology/function [homepage on the Internet]. Oral Presentation. 5th Workshop on Children and Adolescents HIV Exposed and Uninfected; 2019 Jul 21–24; Mexico City [cited n.d.]. Available from: https://www.iasociety.org/HIV-Programmes/Programmes/Paediatrics-CIPHER/5th-HIV-Exposed-Uninfected-Child-and-Adolescent-Workshop.

[28] Marchant A Maternal determinants of immunity in children HIV exposed and uninfected [homepage on the Internet]. Oral Presentation. 5th Workshop on Children and Adolescents HIV Exposed and Uninfected; 2019 Jul 21–24; Mexico City [cited n.d.]. Available from: https://www.iasociety.org/HIV-Programmes/Programmes/Paediatrics-CIPHER/5th-HIV-Exposed-Uninfected-Child-and-Adolescent-Workshop.

[29] Evans C Child mortality growth and early childhood development – Comparing children who are HIV exposed and uninfected and children HIV unexposed in the SHINE trial [homepage on the Internet]. Oral Presentation. 5th Workshop on Children and Adolescents HIV Exposed and Uninfected; 2019 Jul 21–24; Mexico City [cited n.d.]. Available from: https://www.iasociety.org/HIV-Programmes/Programmes/Paediatrics-CIPHER/5th-HIV-Exposed-Uninfected-Child-and-Adolescent-Workshop.

[30] Prendergast A Infant feeding and WASH interventions among children HIV exposed uninfected in SHINE [homepage on the Internet]. Oral Presentation. 5th Workshop on Children and Adolescents HIV Exposed and Uninfected; 2019 Jul 21–24; Mexico City [cited n.d.]. Available from: https://www.iasociety.org/HIV-Programmes/Programmes/Paediatrics-CIPHER/5th-HIV-Exposed-Uninfected-Child-and-Adolescent-Workshop.

[31] Mulenga P, Mutanga J Follow-up of children HIV exposed uninfected through routine child health services: Zambian perspective [homepage on the Internet]. Oral Presentation. 5th Workshop on Children and Adolescents HIV Exposed and Uninfected; 2019 Jul 21–24; Mexico City [cited n.d.]. Available from: https://www.iasociety.org/HIV-Programmes/Programmes/Paediatrics-CIPHER/5th-HIV-Exposed-Uninfected-Child-and-Adolescent-Workshop.

[32] Bamford L, Feucht U Providing care for children HIV exposed uninfected through routine child health services: South African perspective [homepage on the Internet]. Oral Presentation. 5th Workshop on Children and Adolescents HIV Exposed and Uninfected; 2019 Jul 21–24; Mexico City [cited n.d.]. Available from: https://www.iasociety.org/HIV-Programmes/Programmes/Paediatrics-CIPHER/5th-HIV-Exposed-Uninfected-Child-and-Adolescent-Workshop.

[33] Penazzato M Integrating children HIV exposed and uninfected into the nurturing care framework [homepage on the Internet]. Oral Presentation. 5th Workshop on Children and Adolescents HIV Exposed and Uninfected; 2019 Jul 21–24; Mexico City [cited n.d.]. Available from: https://www.iasociety.org/HIV-Programmes/Programmes/Paediatrics-CIPHER/5th-HIV-Exposed-Uninfected-Child-and-Adolescent-Workshop.

